# Association between probiotic and yogurt consumption and kidney disease: insights from NHANES

**DOI:** 10.1186/s12937-016-0127-3

**Published:** 2016-01-27

**Authors:** Rabi Yacoub, Deepak Kaji, Shanti N. Patel, Priya K. Simoes, Deepthi Busayavalasa, Girish N. Nadkarni, John C. He, Steven G. Coca, Jaime Uribarri

**Affiliations:** 1 Department of Medicine, Division of Nephrology, University at Buffalo, Buffalo, NY USA; 2 Department of Medicine, Division of Nephrology, Icahn School of Medicine at Mount Sinai, New York, NY USA; 3 Department of Medicine, Division of Gastroenterology and Clinical Nutrition, Memorial Sloan Kettering Cancer Center, New York, NY USA

**Keywords:** Yogurt, Probiotics, Chronic kidney disease, Proteinuria, NHANES

## Abstract

**Background:**

Data from experimental animals suggest that probiotic supplements may retard CKD progression. However, the relationship between probiotic use, frequent yogurt consumption (as a natural probiotic source), and kidney parameters have not been evaluated in humans.

**Findings:**

We utilized NHANES data, and analyzed the association of probiotic alone (1999–2012) and yogurt/probiotic (2003–2006) use with albuminuria and eGFR after adjustment for demographic and clinical parameters. Frequent yogurt consumption was defined as thrice or more weekly over the year prior to the interview. Frequent yogurt/probiotic consumers had lower adjusted odds of developing combined outcome (albuminuria and/or eGFR < 60 ml/min/1.73 m^2^) compared to infrequent consumers (OR = 0.76; 95 % CI = 0.61-0.94). When evaluated separately, frequent consumers had lower odds of albuminuria and nonsignificant trend towards decreased odds of low eGFR compared to infrequent consumers. In the probiotic cohort, probiotic consumers were found to have a lower adjusted odds of albuminuria compared to nonusers (OR = 0.59; 95 % CI = 0.37–0.94).

**Conclusion:**

Frequent yogurt and/or probiotics use is associated with decreased odds of proteinuric kidney disease. These hypothesis-generating results warrant further translational studies to further delineate the relationship between yogurt/probiotics with kidney dysfunction, as well as microbiome and dysbiosis as potential mediators.

**Electronic supplementary material:**

The online version of this article (doi:10.1186/s12937-016-0127-3) contains supplementary material, which is available to authorized users.

## Findings

### Introduction

The human gastrointestinal tract is a complex ecosystem with 10–100 trillion microorganisms (gut microbiota) [1], that play a major role in the body’s biochemical activities. Studies have demonstrated that gut microbiota can influence numerous aspects of human biology, and alterations in its function and composition (dysbiosis) have been suggested to play a role in pathogenesis of diverse human illnesses such as chronic inflammation, diabetes mellitus, obesity, and cardiovascular diseases [2, 3]. Recently, there is a growing body of evidence that dysbiosis occurs in patients with chronic kidney disease (CKD) leading to continued inflammation, and worsening cardiac and renal dysfunction [4, 5]. Thus, it is possible that affecting this dysbiosis through probiotic interventions could impact renal function.

The United States Food and Drug Administration (FDA) defines yogurt as food produced by culturing dairy ingredient with lactic acid-producing bacteria. To meet the National Yogurt Association's criteria for “live and active culture yogurt,” the finished product must contain at least 10^8^ bacterial organisms per gram [6], making it the most commonly used source of probiotics. Some studies have shown favorable clinical outcomes of yogurt consumption probably through its effect on the gut microbiota [7]. We hypothesized that frequent yogurt consumption would be associated with better kidney parameters.

### Methods

#### Study population and survey

This study utilized cross-sectional data from the 1999–2012 US National Health and Nutrition Survey (NHANES). We utilized the Food Frequency Questionnaire (FFQ, 2003–2006), and Dietary Supplement Use 30-Day (DSQ, 1999–2012) to evaluate both yogurt and probiotic consumption in participants ≥18 years and its association with renal function and disease states. The NHANES program is approved by the NHANES Institutional Review Board (IRB), and the NCHS Research Ethics Review Board (ERB) (after 2003).

We extracted frequency of yogurt use from the FFQ. We divided yogurt consumers based on their weekly consumption into “frequent”, defined as three times or higher weekly, and “infrequent”, defined as less than three times weekly. We extracted probiotic use from the DSQ using text search terms. Thus, we had two distinct cohorts; one with data on probiotic use from 1999 to 2012 (referred to as P cohort) and another one with both yogurt/probiotic use from 2003 to 2006 (referred to as YP cohort).

We calculated estimated glomerular filtration rate (eGFR) using the Chronic Kidney Disease Epidemiology Collaboration (CKD-EPI) formulae. We defined an eGFR-based outcome of eGFR < 60 ml/min/1.73 m^2^ and albuminuria-based outcome of urine albumin creatinine ratio (UACR) > 30 mcg/mg based on previously established cutoffs.

#### Statistical analysis

Continuous variables were analyzed using analysis of variance and *t* test when appropriate, and categorical variables using chi-square test. We evaluated the association between frequent yogurt consumers in the YP cohort and probiotic use in the P cohort with the eGFR and albuminuria outcomes individually and as a composite (albuminuria or eGFR < 60 ml/min/1.73 m^2^) using multivariable logistic regression models after adjusting for confounding factors we determined *apriori* (Table 1 footnote). We performed all analyses using SPSS® (2012. IBM SPSS Statistics for Windows, Version 21.0. Armonk, NY: IBM Corp).

**Table 1:** Association of yoghurt/probiotic and probiotic alone with renal parameters

	Yoghurt and probiotics				Probiotics alone			
	Unadjusted OR (95 % CI)	*P*	Adjusted OR (95 % CI)	*P*	Unadjusted OR (95 % CI)	*P*	Adjusted OR (95 % CI)	*P*
All CKD	0.72(0.60–0.87)	<0.001	0.76 (0.61–0.94)	0.01	0.89 (0.64–1.25)	0.51	0.74 (0.51–1.08)	0.12
Albuminuria	0.65(0.51–0.83)	<0.001	0.74 (0.57–0.95)	0.02	0.63 (0.40–0.98)	0.04	0.59 (0.37–0.94)	0.03
CKDIII and above	0.82(0.65–1.04)	0.10	0.84 (0.62–1.12)	0.22	1.29 (0.86–1.94)	0.22	0.95 (0.59–1.54)	0.84

Models adjusted for age, gender, race, hypertension, diabetes mellitus, body mass index, glycosylated hemoglobin A1C, socioeconomic status (defined as poverty income ratio), duration of probiotic/yoghurt use and medications(Angiotensin Converting Enzyme Inhibitor; Angiotensin Receptor Blocker; Statins and Insulin)

### Results

Of the 41,243 adult participants, complete covariate data was available for 6853 in the YP cohort (2003–2006) and 32,749 in the P cohort (1999–2012). In YP cohort, frequent consumers were of similar age as infrequent consumers, but with better socioeconomic status, more females and non-African Americans, and with less comorbid conditions. While probiotic users in the P cohort were older, they were of better socioeconomic status compared to nonusers, with higher usage frequencies among females and non-African Americans but with no significant differences in the comorbid conditions (Additional file 1: Table S1).

In the YP cohort (2003–2006), 1316(19.2 %) participants were found to have the combined outcome (eGFR < 60 ml/min/1.73 m^2^ or albuminuria). FYP consumers had lower adjusted odds of developing combined outcome compared to infrequent consumers (OR = 0.76; 95 % CI = 0.61–0.94). When we evaluated the association in regards to albuminuria and low eGFR separately, 803 (11.7 %) of participants had detectable albuminuria (Median = 66.5, IQR = 41.4–156.2). FYP consumers had lower odds of albuminuria compared to infrequent consumers (OR = 0.74; 95 % CI = 0.57–0.95). There was a nonsignificant trend towards decreased odds of low eGFR among FYP consumers compared to infrequent consumers (Table 1). Participants frequently consuming yogurt/probiotics were found to have less urinary albumin excretion when compared to infrequent consumers. This trend was statistically insignificant due to the non-normality in distribution, when the logarithmic values were analyzed a statistically significant difference was found between the groups (Additional file 2: Table S2). No difference was found in the estimated glomerular filtration rate between the two groups. To further confirm these findings, we compared the frequent consumers with a subgroup of infrequent consumers (those consuming yogurt less than once a month) and found similar results (aOR = 0.73; 95 % CI = 0.59–0.98, and a OR = 0.8; 95 % CI = 0.65–0.98 for albuminuria and all CKD respectively). No statistically significant difference was found in regards to CKD stage III and above.

When we evaluated association of probiotic use alone with the above-mentioned renal outcomes, probiotic users were found to have a lower adjusted odds of albuminuria compared to nonusers (OR = 0.59; 95 % CI = 0.37–0.94), although there was no association with low eGFR (Table 1).

### Discussion

Using NHANES data we found an inverse association of frequent yogurt/probiotic consumptionand albuminuria in a large, nationally representative general population sample. The associations remained after adjustment for several important predictors and confounders. Previous studies have evaluated the effects of probiotics on renal function and the progression of renal disease by performing short term randomized trials and demonstrated decreased blood urea nitrogen (BUN) levels [8]. Moreover, these studies demonstrated that probiotics decreased inflammatory markers and increased antioxidants that suggest a possible mechanism for beneficial effects of probiotic use. In CKD, dysbiosis and translocation of gut microbiota may occur leading to a continuous inflammatory status [4, 5]. At the same time, certain bacterial products, such as trimethylamine N-oxide [5], p-cresol [9], and indoxyl sulfate [10] have been reported to have direct toxic effects on podocytes and renal tubules through different mechanisms.

Limitations of our study include a relatively small sample size due to the infrequent probiotic supplement usage, thus the trends found in our analysis for less risk of low eGFR might have been statistically significant in a larger sample size. Since our analysis is cross-sectional in nature, we cannot deduce temporality or causation. Though we cannot discount the healthy-user effect (frequent yogurt consumers follow an overall better and more balanced dietary habits and are healthier and have less dietary restrictions compared to infrequent consumers) as a possible confounding factor, we have adjusted for socioeconomic status, comorbid and demographic factors in an attempt to account for it. Another limitation is since NHANES is largely comprised of healthy individuals, the frequency and severity of albuminuria and low eGFR is low, thus it is difficult to show strong associations between yogurt and probiotic consumption due to ceiling effects and narrow range of pathology. Thus, these findings are merely hypothesis-generating results and further translational studies should be performed to further delineate the relationship between yogurt/probiotics and kidney dysfunction, as well as microbiome and dysbiosis as potential mediators.

### Availability of supporting data

The data sets supporting the results of this article are publicly available in the NHANES (National Health and Nutrition Examination Survey) repository available at http://www.cdc.gov/nchs/nhanes.htm. The authors accessed this publicly available data sets and retrieved and merged files in accordance with the NHANES guidelines and recommendations.

## Additional files

Additional file 1: Table S1. Baseline Characteristics of NHANES sub cohort stratified by yoghurt/probiotic use. (DOC 39 kb)

Additional file 2: Table S2. UAC and eGFR between frequent and infrequent consumers. (DOC 27 kb)

## Abbreviations

NHANES: National health and nutrition survey
eGFR: estimated Golmerular Filtration Rate
CKD: Chronic kidney disease
FDA: The United States Food and drug administration
FFQ: Food frequency questionnaire
DSQ: Dietary supplement use
YP cohort: Yogurt/probiotic use cohort (2003–2006)
P cohort: Probiotic use cohort (1999–2012)
FYP: Frequent yogurt/probiotic consumers
BUN: Blood urea nitrogen
ACEi: Angiotensin converting enzyme inhibitor
ARB: Angiotensin receptor blocker

## References

[1] Cani PD, Delzenne NM (2009). The role of the gut microbiota in energy metabolism and metabolic disease. Curr Pharm Des.

[2] Nicholson JK, Holmes E, Kinross J, Burcelin R, Gibson G, Jia W (2012). Host-gut microbiota metabolic interactions. Science.

[3] Ebel B, Lemetais G, Beney L, Cachon R, Sokol H, Langella P (2014). Impact of probiotics on risk factors for cardiovascular diseases. A review. Crit Rev Food Sci Nutr.

[4] Vaziri ND, Wong J, Pahl M, Piceno YM, Yuan J, DeSantis TZ (2013). Chronic kidney disease alters intestinal microbial flora. Kidney Int.

[5] Tang WH, Wang Z, Kennedy DJ, Wu Y, Buffa JA, Agatisa-Boyle B (2015). Gut microbiota-dependent trimethylamine N-oxide (TMAO) pathway contributes to both development of renal insufficiency and mortality risk in chronic kidney disease. Circ Res.

[6] Adolfsson O, Meydani SN, Russell RM (2004). Yogurt and gut function. Am J Clin Nutr.

[7] Pei R, Martin DA, DiMarco DM, Bolling BW. Evidence for the Effects of Yogurt on Gut Health and Obesity. Crit Rev Food Sci Nutr 2015:0. Epub ahead of print 10.1080/10408398.2014.883356 25875150

[8] Ranganathan N, Friedman EA, Tam P, Rao V, Ranganathan P, Dheer R (2009). Probiotic dietary supplementation in patients with stage 3 and 4 chronic kidney disease: a 6-month pilot scale trial in Canada. Curr Med Res Opin.

[9] Watanabe H, Miyamoto Y, Honda D, Tanaka H, Wu Q, Endo M (2013). p-Cresyl sulfate causes renal tubular cell damage by inducing oxidative stress by activation of NADPH oxidase. Kidney Int.

[10] Ichii O, Otsuka-Kanazawa S, Nakamura T, Ueno M, Kon Y, Chen W (2014). Podocyte injury caused by indoxyl sulfate, a uremic toxin and aryl-hydrocarbon receptor ligand. PLoS One.

