# Supplementary material for: Association between probiotic and yogurt consumption and kidney disease: insights from NHANES
Source: Nutr J. 2016 Jan 27;15:10. doi: 10.1186/s12937-016-0127-3 (PMC4728789; doi:10.1186/s12937-016-0127-3)
Supplement: Supplementary file 1 — Baseline Characteristics of NHANES sub cohort stratified by yoghurt/probiotic use. (DOC 39 kb) [file 12937_2016_127_MOESM1_ESM.doc]

**Additional file 1 Table S1:** Baseline Characteristics of NHANES sub cohort stratified by yoghurt/probiotic use

| **Variables** | **Yogurt/Probiotic Cohort (2003-2006)** | | | | **Probiotic Cohort (1999-2012)** | | | |
| --- | --- | --- | --- | --- | --- | --- | --- | --- |
|  | All  N= 6853 | Frequent  N= 969 | Infrequent  N= 5884 | P | All  N= 32749 | Users  N= 270 | Nonusers  N= 32479 | P |
| **Age** | 48.2±20.1 | 48.4±19.1 | 48.1±20.2 | 0.63 | 46.69±19.3 | 53.37±18 | 46.63±19.3 | <0.01 |
| **Male-%** | 45.2 | 29.7 | 47.8 | <0.001 | 48.4 | 33.7 | 48.6 | <0.001 |
| **African Americans-%** | 19.8 | 13.2 | 20.9 | <0.001 | 20.7 | 11.9 | 20.7 | <0.01 |
| **Hypertension-%** | 32.5 | 28.5 | 33.1 | 0.002 | 30.9 | 30 | 30.1 | 0.39 |
| **Diabetes Mellitus-%** | 11 | 8.3 | 11.4 | 0.002 | 11.4 | 8.9 | 11.4 | 0.12 |
| **ACEi/ARB-%** | 16.5 | 13.4 | 17 | 0.003 | 16 | 15.2 | 16 | 0.39 |
| **Statin Use-%** | 12.9 | 11.5 | 13.1 | 0.08 | 12.3 | 10.4 | 12.3 | 0.19 |
| **Insulin Use-%** | 2.1 | 1.4 | 2.2 | 0.08 | 2.1 | 0.7 | 2.1 | 0.07 |
| **Poverty Income Ratio** | 2.65±1.6 | 3.05±1.7 | 2.58±1.6 | <0.001 | 2.51±1.6 | 3.14±1.7 | 2.51±1.6 | <0.01 |
| **Hemoglobin A1c** | 5.54±0.9 | 5.41±0.8 | 5.56±0.9 | <0.001 | 5.62±1 | 5.48±0.8 | 5.62±1 | 0.03 |
| **Body-mass index** | 28.5±6.6 | 28.1±6.5 | 28.5±6.7 | 0.07 | 28.51±6.6 | 26.8±5.8 | 28.5±6.6 | <0.01 |
| **Systolic Blood Pressure** | 123±20 | 121±19 | 124±20 | <0.001 | 123±19 | 122±18 | 123±19 | 0.6 |
| **Diastolic Blood Pressure** | 68±13.6 | 68±12 | 68±14 | 0.78 | 69±13 | 70±12 | 69±13 | 0.41 |

All plus-minus values are Means±Standard Deviations. ACEi=Angiotensin Converting Enzyme Inhibitor; ARB=Angiotensin Receptor Blocker; Body-mass Index was calculated as weight in kg divided by the square of height in meters; Blood pressures are in mm of Hg
