# Supplementary material for: Association between probiotic and yogurt consumption and kidney disease: insights from NHANES
Source: Nutr J. 2016 Jan 27;15:10. doi: 10.1186/s12937-016-0127-3 (PMC4728789; doi:10.1186/s12937-016-0127-3)
Supplement: Supplementary file 2 — UAC and eGFR between frequent and infrequent consumers. (DOC 27 kb) [file 12937_2016_127_MOESM2_ESM.doc]

**Additional file 2: Table S2: UAC and eGFR between frequent and infrequent consumers**

|  | Frequent users | Infrequent users | P |
| --- | --- | --- | --- |
| UAC (Mean; 95% CI) | 34.8 (18.6-50.9) | 39.2 (32-46.4) | 0.6 |
| LnUAC (Mean; ; 95% CI) | 2.09 (2.02-2.16) | 2.18 (2.14-2.21) | 0.03 |
| eGFR (Mean; ; 95% CI) | 93.4 (91.8-94.9) | 93.1 (92.4-93.7) | 0.7 |

UAC: Urine albumin creatinine ratio; CI: Confidence interval; eGFR: Estimated glomerular filtration rate
